# Immunohistochemical Characterisation of the Interstitial Inflammatory Environment: T-Cell- and B-Cell-Dominant Subtypes of Hidradenitis Suppurativa

**DOI:** 10.3390/dermatopathology12030025

**Published:** 2025-08-25

**Authors:** Nessr Abu Rached, Stefanie Bruckmüller, Martin Doerler, Hanna Telkemeyer, Lennart Ocker, Yannik Haven, Daniel Myszkowski, Markus Stücker, Eggert Stockfleth, Falk G. Bechara

**Affiliations:** 1 International Centre for Hidradenitis Suppurativa/Acne Inversa (ICH), Department of Dermatology, Venereology and Allergology, Ruhr-University Bochum, 44791 Bochum, Germany; stefanie.brueckmueller@kklbo.de (S.B.); martin.doerler@kklbo.de (M.D.); hanna.telkemeyer@kklbo.de (H.T.); lennart.ocker@kklbo.de (L.O.); daniel.myszkowski@kklbo.de (D.M.); markus.stuecker@kklbo.de (M.S.); eggert.stockfleth@kklbo.de (E.S.); falk.bechara@kklbo.de (F.G.B.); 2 Skin Cancer Center, Department of Dermatology, Venereology and Allergology, Ruhr-University Bochum, 44791 Bochum, Germany

**Keywords:** hidradenitis suppurativa, lymphocytes, inflammation, inflammatory environment, T-cells, B-cells, plasma cells

## Abstract

Background: Hidradenitis suppurativa (HS) is a chronic inflammatory disease with a complex immune response. Given the considerable heterogeneity of the clinical phenotype of HS, this study aimed to analyse the immunohistochemical pattern of interstitial inflammation. Methods: Immunohistochemical analysis was performed on skin samples from 49 patients with HS. The immunohistochemical markers CD3, CD4 and CD8 for T-cells, CD20 for B-cells, CD138 for plasma cells and CD30, CD56, Bcl-2 and Bcl-6 were stained on lesional skin. Results: The analysis of immune cell dominance in patients with HS revealed that 33.3% of the cohort exhibited B-cell dominance, defined as a ratio of the sum of CD20+ and CD138+ cells to CD3+ cells greater than 1, while the majority (66.7%) demonstrated T-cell dominance, defined as a ratio of CD3+ cells to the sum of CD20+ and CD138+ cells greater than 1. B-cell-dominant HS is associated with a significantly elevated probability of mammary involvement (13.3% vs. 0%; *p* = 0.041). T-cell-dominant HS patients tended to demonstrate a higher mean tobacco consumption, but not significantly (20 vs. 5 tobacco pack-years; *p* = 0.06). CD4-dominant HS patients exhibited a significantly greater involvement of the mons pubis (62.5% vs. 28.6%, *p* = 0.023) compared to CD8-dominant patients, who demonstrated a significantly higher number of abscesses (*p* = 0.027). Conclusions: For the first time, we describe the clinical and immunohistochemical characteristics of T-cell- and B-cell-dominant HS. Although HS seems to be more dominated by T-cells, a B-cell dominance was found in 33% of cases.

## 1. Introduction

Hidradenitis suppurativa (HS) is a chronic inflammatory skin disease characterised by painful, recurrent nodules, abscesses, and draining fistula tracts, mainly affecting the intertriginous areas. HS patients have the highest inflammatory levels of all dermatological patients, demonstrating that the inflammatory process is central to HS [1,2,3]. Despite its clinical significance and high incidence, the pathogenesis of HS is not fully understood [4]. HS is increasingly treated with multimodal approaches. The multimodal approach includes biologics, antibiotics, supportive care (lifestyle management, pain management and psychosocial care) and surgery. Three biologics are currently approved in Europe for the treatment of moderate to severe HS. HS is currently classified as an autoimmune disease, which can affect various organs [5,6,7,8]. While it is known that HS is associated with an aberrant immune response, the specific contributions of different immune cell subsets, particularly T and B lymphocytes, to the interstitial inflammatory milieu are not fully understood.

T lymphocytes (T-cells) and B lymphocytes (B-cells) play a crucial role in adaptive immunity, with T-cells predominantly involved in cell-mediated responses and B-cells responsible for antibody production [9]. In HS, emerging evidence suggests that the infiltration of lymphocytes into the skin contributes to the chronic inflammation and tissue remodelling, although data for detailed immunohistochemical characterisation of these lymphocytes are limited. Recent findings suggest the presence of tertiary lymphoid structures within HS lesions and persistent cutaneous B-cell development [10]. Specifically, B-cell activating factor (BAFF) is involved in the persistence and function of B-cells and plasma cells in HS lesions [11]. Therefore, B-cell-modulating therapies for HS are increasingly being investigated.

The aim of this study is to provide a comprehensive immunohistochemical analysis of the interstitial inflammatory milieu in HS, focusing on the presence and distribution of T and B lymphocyte subsets. Specifically, the aim was to analyse the clinical data (personal and disease-specific factors) with the results of the inflammatory microenvironment.

## 2. Materials and Methods

### 2.1. Design and Setting

We retrospectively included skin tissue from 49 patients with confirmed HS from the International Centre for Hidradenitis suppurativa/Acne inversa (ICH) at the Ruhr University Bochum, Germany. This study was conducted in accordance with the Declaration of Helsinki (12). The present retrospective study was approved by the Institutional Ethics Committee of the Ruhr University Bochum (ethics vote: #5302/15).

### 2.2. Antibodies

The antibodies used in this study were as follows: CD3 (rabbit polyclonal, ready-to-use, Agilent Dako, code no. A0452), CD4 (mouse monoclonal, clone 4B12, ready-to-use, Agilent Dako, code no. IR649), CD8 (mouse monoclonal, clone C8/144B, ready-to-use, Agilent Dako, code no. IR623), CD20 (mouse monoclonal, clone L26, ready-to-use, Agilent Dako, code no. IR604), CD30 (mouse monoclonal, clone Ber-H2, ready-to-use, Agilent Dako, code no. IR602), CD56 (mouse monoclonal, clone 123C3, ready-to-use, Agilent Dako, code no. IR628), CD138 (mouse monoclonal, clone MI15, ready-to-use, Agilent Dako, code no. GA642), Bcl-2 (mouse monoclonal, clone 124, ready-to-use, Agilent Dako, code IR614) and Bcl-6 (mouse monoclonal, clone PG-B6p, ready-to-use, Agilent Dako, code IR625). All antibodies were processed using Tris-EDTA buffer (pH 9) and incubated for 30 min at room temperature.

### 2.3. Skin Samples, Staining and Quantitative Analysis of Immunohistochemistry

Initially, archived haematoxylin–eosin (HE) stains were used to search for inflammation in skin tissue. Only HE specimens with present inflammation were included. Thus, specimens with only chronic fibrosis and no inflammation were excluded. Skin samples from 49 HS patients were analysed and specifically stained for T-cell (CD3, CD4, CD8), B-cell (CD20) and plasma cell (CD138) markers, as well as other markers such as CD30 (expression in activated T-cells and B-cells), CD56 (expression of natural killer cells), Bcl-2 (expression in B and T-cells and promotes anti-apoptosis) and Bcl-6 (expression in activated germinal centre B-cells and T follicular helper cells). Skin tissue from surgical excisions was used in this investigation. Formalin-fixed paraffin embedded tissue was cut at 4 µm and transferred to coated slides. Immunohistological staining of tissue sections was performed according to the protocol of the DAKO REAL™ Detection System, Alkaline Phosphatase/RED, Rabbit/Mouse (Code No. K5005). Image analysis was semi-automated using a NanoZoomer S60 slide scanner from Hamamatsu. Counting of positively stained cells in the five fields of view was performed using the open-trace bioimage analysis software Qupath (400× magnification; version 0.5.1, 2024, Edinburgh, Scotland) [12,13]. Immunohistochemistry using the QuPath programme is validated and reproducible [14]. Colour intensity was graded by Qupath as weak, moderate and strong staining. The *H*-score was calculated from the above values using the Qupath programme according to the following formula [15]:

*H*-score = (1 × percentage of weak staining) + (2 × percentage of moderate staining) + (3 × percentage of strong staining); (1)

B-cell dominance was present if the percentage of the sum of CD20+ and CD138+ cells was higher than the percentage of CD3-positive cells. T-cell dominance was present if the percentage of CD3-positive cells was greater than that of the sum of CD20+ and CD138+ cells. CD8 dominance was present with a ratio of CD4/CD8 < 1 and CD4 dominance with a ratio of CD4/CD8 > 1.

### 2.4. Patients and Data Collection

Retrospective data on personal factors (sex, BMI, age, comorbidities, smoking status, tobacco pack-years, etc.) and disease-specific factors (severity of disease, anatomic localisation, age of onset, duration of disease) were collected from patient records. Only patients with HS for whom all of the parameters were available were included. Patients’ HS disease severity was classified according to the modified Hidradenitis Suppurativa Score (mHSS), Hurley system and Severity Assessment of Hidradenitis Suppurativa (SAHS) [16,17,18].

### 2.5. Data Analysis

The study data were analysed using a variety of statistical methods. Results are presented as percentages, means with standard deviations (mean ± SD) or medians with interquartile ranges (IQR), depending on the type of data. Normally distributed data are presented as means with standard deviations (mean ± SD) and non-normally distributed data are presented as medians with interquartile ranges or ranges. To ensure the appropriate use of these descriptive statistics, the normality of the data distribution was carefully checked using the Shapiro–Wilk test and visualised using Q-Q plots. Descriptive statistics were employed to summarize the demographic and clinical characteristics of the patients, ensuring a clear and comprehensive overview of the study population. The independent *t*-test for normally distributed continuous data and the Mann–Whitney U-test for non-normally distributed data were used to determine mean differences. For binary data, the Chi-square test was used.

Statistical analysis was conducted using IBM SPSS Statistics version 29.0 (IBM Corporation, Armonk, NY, USA), with the significance level set at *p* < 0.05.

## 3. Results

### 3.1. Patients’ History and Basic Characteristics

Table 1 summarizes the characteristics of 49 patients with HS. Among them, 18 (36.7%) were female and 31 (63.3%) male, with a median age of 48 years (IQR 35–57), and the median age of HS onset was 27 years (IQR 19–37). The median HS duration was 12 years (IQR 7–24), and the median BMI was 31.4 kg/m^2^ (IQR 28.3–36.5). Family history for HS was positive in 14 (28.6%) cases. Smoking status showed 33 (67.3%) active smokers, 12 (24.5%) non-smokers and 4 (8.2%) ex-smokers. Disease severity was categorized as Hurley stage I (n = 1, 2.0%), II (n = 20, 40.8%) and III (n = 28, 57.1%). Median mHSS was 49 (IQR 25–89), median SAHS was 8 (IQR 5–9), with median inflammatory nodules at 1 (IQR 0–4), abscesses at 0 (IQR 0–0), flare-ups at 0 (IQR 0–2), fistula tracts at 6 (IQR 3–11) and affected regions at 3 (IQR 2–5). Common comorbidities included obesity (30, 58.8%), hypertension (18, 35.3%), diabetes (10, 17.7%), hypothyroidism (8, 15.7%) and dyslipidaemia (7, 13.7%). Among the 49 patients included in this study, 8 (16.3%) were receiving adalimumab therapy at the time of the examination and 8 (16.3%) of the patients were receiving antibiotic therapy.

### 3.2. Immunohistochemical Results of the Markers CD3, CD4, CD8, CD20, CD30, CD56, CD138, Bcl-2 and Bcl-6

**Figure 1:**
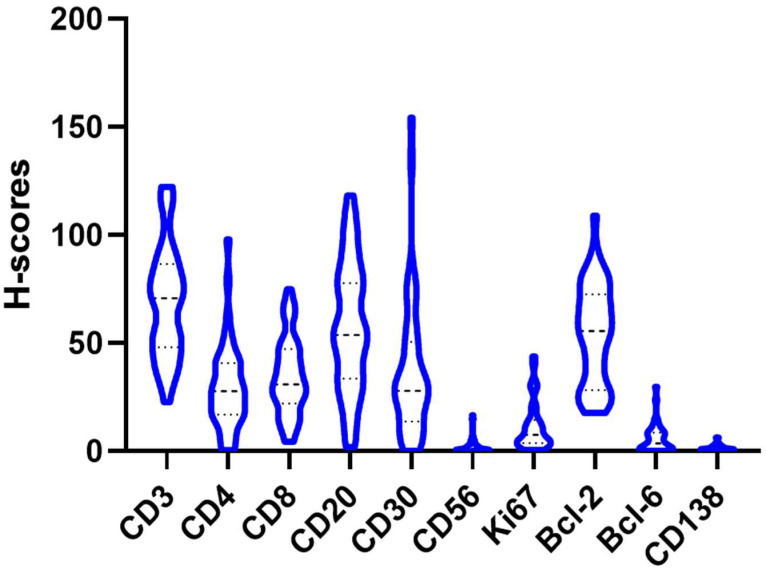
Distribution of the *H*-score of the different antibodies (Bcl-2, Bcl-6, CD3, CD4, CD8, CD20, CD30, CD56 and CD138) in a violin plot.

**Figure 2:**
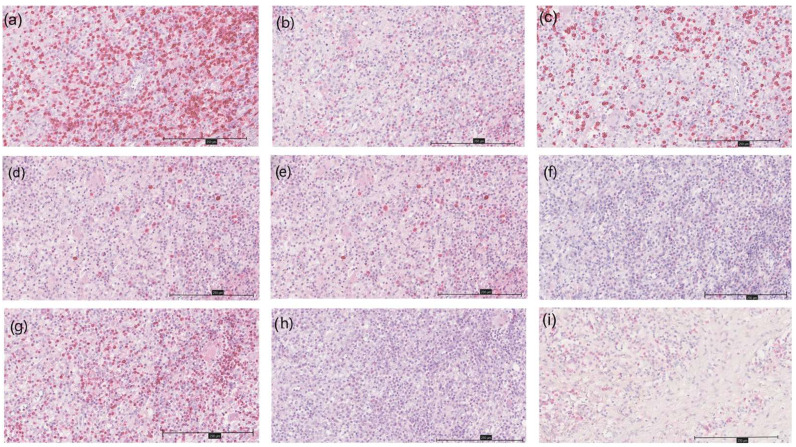
Immunohistochemistry of different markers in a HS patient at 200× magnification; (**a**) CD3; (**b**) CD4; (**c**) CD8; (**d**) CD20; (**e**) CD30; (**f**) CD56; (**g**) Bcl-2; (**h**) Bcl-6; (**i**) CD138.

**Figure 3:**
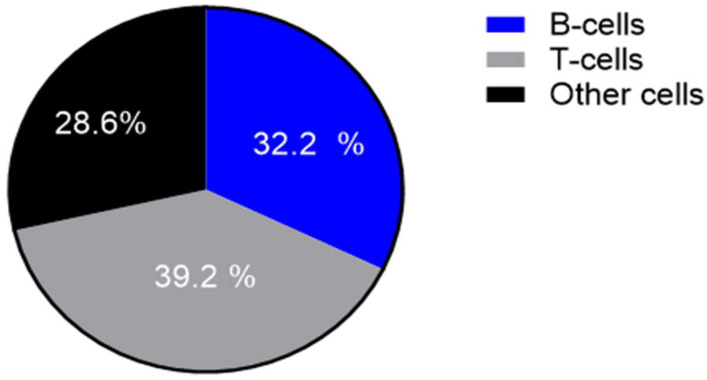
Proportion of T-cells, B-cells (including plasma cells) and other cells (including histiocytes, multinucleated giant cells, neutrophilic granulocytes) in a cake diagram.

Table 2 and Figure 1 and Figure 2 summarize the immunohistochemical analysis of markers CD3, CD4, CD8, CD20, CD30, CD56, CD138, Bcl-2 and Bcl-6 in skin tissue from 49 HS patients. The results are presented as absolute counts of positive cells (mean ± SD), the relative percentage of positive cells (median, IQR) and the *H*-score (mean ± SD). Notably, CD3 exhibited the highest mean absolute count of 375.2 (±176.1) with 39.2% (median, IQR 31.5–47.2) of cells staining positive, and an *H*-score of 66.2 (±28.8). In contrast, CD56 showed the lowest values across all parameters. CD3+ T-cells represented 39.2%, B-cells (including plasma cells) 32.2.% and other inflammatory cells (including histiocytes, giant cells and neutrophils) 28.6% of the tissue analysed (Figure 3).

Appendix A and Appendix B present detailed statistical analyses of different markers and their expression in patients with HSFF based on different variables such as positive family history of HS, disease severity, gender and common comorbidities (obesity, hypertension, diabetes mellitus). No statistically significant differences in marker expression were found between patients with and without a positive family history of HS (*p* > 0.05). There were no differences in marker expression between patients with severe disease (Hurley III) and those with mild to moderate disease (Hurley I and II; *p* > 0.05). A significant difference in Bcl-2 expression was observed, with males having higher levels than females (*p* = 0.017). No other markers showed an association with gender (*p* > 0.05). The CD4/CD8 ratio was significantly higher in obese HS patients compared to non-obese HS patients (*p* = 0.014). The presence of comorbidities such as arterial hypertension and diabetes mellitus showed no difference in the expression of all markers (*p* > 0.05; Appendix A). Treatment with adalimumab or antibiotics had no influence on the different expression of the markers (*p* > 0.05).

### 3.3. CD4 Dominance Is Associated with Increased Mons Pubis Involvement, CD8 Dominance with Increased Abscess Formation

Appendix C provides a detailed analysis of the CD4/CD8 ratio in the lesional skin tissue of HS patients, categorizing them into two groups based on T-cell dominance: CD8 dominance (CD4/CD8 ratio < 1) and CD4 dominance (CD4/CD8 ratio > 1). Of the 45 patients studied, 46.7% had CD8 dominance and 53.3% had CD4 dominance. Notably, CD4-dominant HS patients tended to have a higher BMI (median BMI 35.0 vs. 29.2; *p* = 0.05) and a significantly greater involvement of the mons pubis region (62.5% vs. 28.6%, *p* = 0.023). Conversely, CD8-dominant patients had a significantly higher number of abscesses (*p* = 0.027), suggesting an association between CD8-positive T-cell prevalence and increased abscess formation. Skin-resident memory CD8-positive T-cells may induce the recruitment of neutrophils and explain the increased number of abscesses in CD8-dominant HS [19]. However, no significant differences in overall disease severity, disease duration, treatment regime or comorbidities were observed between the two groups (*p* > 0.05).

### 3.4. T-Cell Dominance Is More Common than B-Cell Dominance and Shows Different Clinical Features

**Figure 4:**
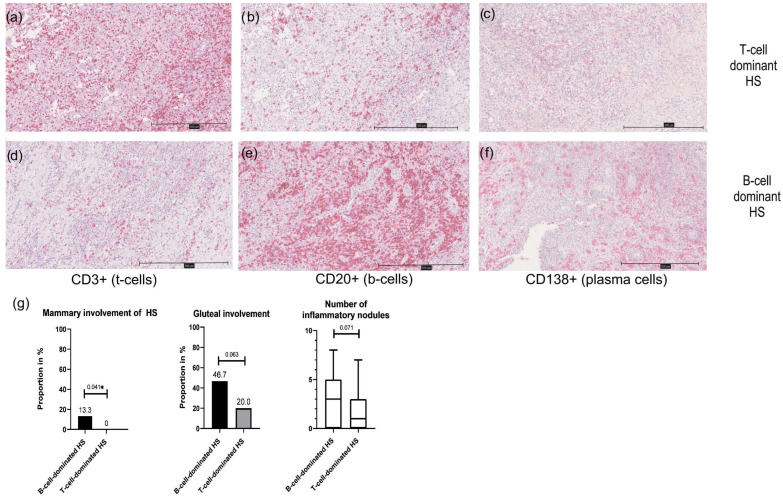
Presentation of a T-cell-dominant HS subtype at 100× magnification (**a**–**c**); (**a**): immunohistochemistry for CD3; (**b**): immunohistochemistry for CD20; (**c**): immunohistochemistry for CD138; expression of CD3+ cells > sum of CD20+ and CD138+ cells) compared to a B-cell-dominant HS subtype at 100× magnification (**d**–**f**); (**d**): immunohistochemistry for CD3; (**e**): immunohistochemistry for CD20; (**f**): immunohistochemistry for CD138; expression of CD3+ cells < sum of CD20+ and CD138+ cells); (**g**): clinical characteristics of B-cell- and T-cell-dominant HS are presented as bar graphs or box plots. B-cell-dominant HS is more likely to show mammary and gluteal involvement and more nodules than T-cell-dominant HS; * significant result.

The analysis of immune cell dominance in HS patients reveals that 33.3% of the cohort exhibited B-cell dominance (sum of CD20+ and CD138+ cells > CD3+ cells), while the majority (66.7%) showed T-cell dominance (CD3+ cells > sum of CD20+ and CD138+ cells). Figure 4 shows the immunohistochemistry of a T-cell-dominated (Figure 4a–c) and a B-cell-dominated HS subtype (Figure 4d–f). Appendix D shows the characteristics of T-cell- and B-cell-dominant HS. Although the demographic and clinical characteristics were largely similar between the two groups, B-cell-dominated patients tended to be younger at disease onset (median age 23 vs. 27.5 years; *p* = 0.2) and had a lower median tobacco pack-years (5 vs. 20 pack-years; *p* = 0.06) though these differences were not statistically significant (*p* > 0.05). Clinically, B-cell dominance was associated with a higher number of inflammatory nodules, but not significantly (*p* = 0.071; Figure 4e). A significantly increased likelihood of mammary involvement (13.3% vs. 0%; *p* = 0.041), suggesting a potential link between B-cell prevalence and specific disease manifestations. Gluteal involvement was more common in the B-cell-dominant group (46.7% vs. 20%), although this difference was also just short of significant (*p* = 0.063). All other parameters (in particular HS severity, BMI, comorbidities, treatment regime and sex) showed no association with B-cell- or T-cell-dominant HS (*p* > 0.05).

## 4. Discussion

Our results demonstrate that HS is a heterogeneous disease with a highly variable pattern of inflammation. Clinical HS manifestation is also very heterogeneous [20,21], so this is also consistent with the heterogeneous microenvironment findings. The inflammatory infiltrate in HS patients consists of about 70% lymphocytes; the other 30% are usually histiocytes, multinucleated giant cells, plasma cells or neutrophilic granulocytes. Most patients (66%) have a T-cell dominance in the inflammatory microenvironment. This suggests a prominent presence of T-cells in the tissue, indicating a significant T-cell-mediated immune response in HS patients.

T-cells can be classified according to their surface molecules. T-cells include CD4-positive cells (T helper cells; e.g., Th1, Th2, Th17 and Treg cells) and CD8-positive cells (cytotoxic cells) [22]. There are already several investigations about T lymphocytes in HS. HS is characterized by a dysregulation of Th17 [23]. A study by Thomi et al. confirmed that HS is a Th1/Th17-mediated inflammatory skin disease [24]. Compared to healthy controls, the frequency of Tregs cells among CD4-positive T lymphocytes in blood was significantly reduced in the HS group [25]. Hessam et al. suggested that Tregs reach the inflammatory hot spots in HS skin and are therefore reduced in the blood [25]. Melnik et al. describe a shift in the Th17/Treg balance towards Th17, which may have a negative impact on Treg-driven hair follicle stem cell homeostasis and infundibular integrity [26].

Interestingly, patients with T-cell-dominant HS showed a tendency towards a higher mean number of tobacco pack-years compared to the B-cell-dominant HS subtype (20 vs. 5 pack-years; *p* = 0.06). Although this difference was not statistically significant (*p* > 0.05), this link is very immunologically interesting. Smoking mainly activates the Th1 and Th17 pathways. Chronic smoking in HS patients may explain the T-cell dominance. For example, the activation of Th1 and Th17 cells in the pathogenesis of smoking-associated diseases such as COPD is well studied. In COPD, activation releases pro-inflammatory cytokines that lead to tissue damage and chronic inflammation [27,28]. Similar processes to those in lung tissue may also occur in the skin as a result of inflammation. Further research in this area is needed.

Our investigation shows that the absolute numbers of CD4-positive (212.4 ± 125.7) and CD8-positive (184.1 ± 117.9) T-cells are relatively close, with CD4 being slightly higher. To further evaluate the result, we calculated the CD4/CD8 ratio. CD4/CD8 ratio is a well-established measure of various diseases, such as HIV (ratio is determined from blood) or sarcoidosis (bronchioalveolar lavage) [29,30,31]. Circulating CD4/CD8 ratio is a prognostic factor for response to total skin electron beam therapy [32]. CD4/CD8 ratio from the skin is still the focus of dermatological research and was investigated in dermatoses [33,34,35,36]. A study by Żychowska et al. showed that the CD4/CD8 ratio in cutaneous lichen planus is 1.75:1 [33]. In psoriasis, the CD4/CD8 ratio of 2.1:1 is also on the side of the CD4-positive cells [34]. A histopathological study by Harvell et al. examined the CD4/CD8 ratio in various dermatoses (psoriasis, spongiotic dermatitis, PLEVA, lichen planus, lichen sclerosus, herpetic dermatitis, erythema annulare centrifugum, urticaria, normal skin) [36]. They found that all CD4/CD8 ratios of dermatological diseases and healthy skin ranged from 1.0 to 6.0, but were never below 1.0 [36]. However, the results in our HS cohort are surprisingly unexpected. Approximately 46% in our HS cohort show CD4/CD8 ratios < 1 and 53% of patients show an elevated ratio (>1). These results also show that the clinical inflammatory pattern of CD4/CD8 is heterogeneous. However, determining the CD4/CD8 ratio may also have therapeutic implications in HS. Currently, no studies have explored the therapeutic significance of the CD4/CD8 ratio in HS. In systemic lupus erythematosus, the CD4/CD8 ratio is associated as a good indicator of therapeutic efficacy [37].

CD20, a B-cell marker, shows considerable variation (mean ± SD: 325.6 ± 279.6), as reflected by the wide interquartile range of the relative percentage (18.3–43%). Recently, the role of B-cells and plasma cells in HS has received increasing research attention [10,11,38]. The low level of CD138+ cells in HS tissue indicates that, although they are present, plasma cells do not constitute the dominant cell population. Ianalumab, which inhibits the interaction between B-cell activating factor (BAFF) and the BAFF receptor, is currently in a phase II trial in HS patients (clinical trial: NCT03827798). In our perspective, we recommend that HS patients in clinical trials be stratified using B-cell markers (e.g., sum of CD20 and CD138) and T-cell markers (e.g., CD3). B-cell-dominant disease may benefit more from the inclusion of B-cell-dominant HS. Clinically, B-cell-dominant HS is associated with a significantly elevated probability of mammary HS involvement. An exact explanation for this link was not identified and should be investigated further. Exploring the potential mechanisms underlying this association would be valuable. Differentiating between B-cell- and T-cell dominance in HS is useful for distinguishing between mild, moderate disease (Hurley stages I and II) and severe disease (Hurley III). This demonstrates the heterogeneity of HS and the usefulness of other markers, such as haptoglobin, for differentiation [2,39].

CD56 and Bcl-6 show very low absolute numbers and *H*-scores, indicating that NK-cells (marked by CD56) and Bcl-6-expressing cells are not major players in the immune landscape of HS-affected skin tissue. Bcl-6 is usually expressed by germinal centre B-cells and follicular helper T-cells [40,41,42]. NK cells play an important role in acute infections, providing a rapid and non-specific immune response against pathogens, for example [43]. The low incidence of NK cells in the microenvironment confirms that chronic inflammation, rather than acute infection, is the predominant factor in HS. Both CD30 (associated with activated T-cells and certain lymphoma types) and Bcl-2 (an anti-apoptotic marker) are moderately expressed. The presence of CD30 could indicate a subset of activated T-cells or the presence of atypical cells, while Bcl-2 expression points towards resistance to apoptosis in certain cell populations within the tissue. Increased Bcl-2 expression was found in male HS patients compared to female HS patients. This association is probably not HS-specific, but gender-specific. In male rats, for example, a sex difference with higher Bcl-2 expression was observed [44]. Testosterone is suspected to have a positive effect on Bcl-2 expression [45]. Further investigations in this field are required.

The limitations of our study are the retrospective design and the associated limitations in causality and selection bias. However, the high number of cases (n = 49) of immunohistological specimens and the correlation of clinical and immunohistological data is a strength of this study. Another limitation is that the T-cells and B-cells were not differentiated into subtypes.

In future clinical studies, it will be important to consider the pattern of inflammation. Before initiating a specific therapy (e.g., B-cell modifying therapy), it may be beneficial to determine the immunohistochemical subtype (T-cell- vs. B-cell-dominant type or CD4- vs. CD8-dominant type). The recently approved drugs secukinumab and bimekizumab for HS target the cytokines IL17A and IL17AF secreted by Th17 cells, a subset of T helper cells. Therefore, it is possible that T-cell-dominant HS subtypes may benefit more than B-cell-dominant HS subtypes. Further studies are needed in this area.

## 5. Conclusions

Overall, the immunohistochemical profile suggests a complex immune milieu in HS, with significant involvement of T-cells, a variable B-cell response and low levels of NK-cells. For the first time, we describe the clinical and immunohistochemical characteristics between T-cell- and B-cell-dominant HS. HS is a predominantly T-cell-dominant disease. Nevertheless, HS is B-cell-dominant in 33% of cases. Therefore, immunohistochemical phenotyping is of great importance for the development of future therapeutic strategies and prior to the introduction of specific B-cell immunomodulators.

## Figures and Tables

**Table 1:** Personal characteristics and disease-specific characteristics of patients with HS ^a^.

Parameters	Value
Sex, n (%)	
Female	18 (36.7)
Male	31 (63.3)
Age, median (IQR), y	48 (35–57)
Age of HS onset, median (ICR), y	27 (19–37)
Duration of HS, median (ICR), y	12 (7–24)
BMI, median (IQR), kg/m^2^	31.4 (28.3–36.5)
Family history of HS, n (%)	
Positive	14 (28.6)
Negative	35 (71.4)
Smoking status, n (%)	
Active smokers	33 (67.3)
Non-smokers	12 (24.5)
Ex-smoker	4 (8.2)
Tabacco pack-years, median (ICR), y	20 (0–30)
Hurley stage, n (%)	
Hurley I	1 (2.0)
Hurley II	20 (40.8)
Hurley III	28 (57.1)
mHSS, median (IQR)	49 (25–89)
SAHS, median (IQR)	8 (5–9)
Number of inflammatory nodules, median (IQR)	1 (0–4)
Number of abscesses, median (IQR)	0 (0–0)
Flare-ups in the last 4 weeks, median (IQR)	0 (0–2)
Number of fistula tracts, median (IQR)	6 (3–11)
Number of affected regions, median (IQR)	3 (2–5)
Current pain on a visual analogue scale, median (IQR)	3 (0–6)
Ongoing therapy with adalimumab	8 (16.3)
Ongoing therapy with antibiotics	8 (16.3)
Comorbidities ^b^, n (%)	
Obesity	30 (58.8)
Hypertension	18 (35.3)
Diabetes mellitus	10 (17.7)
Hypothyroidism	8 (15.7)
Dyslipidaemia	7 (13.7)

Abbreviations: n, absolute number of patients; y, years; BMI, body mass index; IQR, interquartile range; mHSS, modified Hidradenitis Suppurativa Score; HS, Hidradenitis suppurativa; SAHS, Severity Assessment of Hidradenitis Suppurativa; mHSS, modified Hidradenitis Suppurativa Score. ^a^ Total number of HS patients: 49; ^b^ 5 most common comorbidities in our cohort.

**Table 2:** Immunohistochemical results of the markers CD3, CD4, CD8, CD20, CD30, CD56, CD138, Bcl-2 and Bcl-6 ^a^.

Parameter	Absolute Positive Cells, Mean (±SD)	Relative Number of Positive Cells in %, Median (IQR)	*H*-score, Mean (±SD)
Immunohistochemical markers			
CD3	375.2 (±176.1)	39.2 (31.5–47.2)	66.2 (±28.8)
CD4	212.4 (±125.7)	22.4 (14.4–28.9)	28.8 (±19.5)
CD8	184.1 (±117.9)	18 (8.6–24.7)	31.7 (±19.6)
CD20	325.6 (±279.6)	30.7 (18.3–43)	53.8 (±32)
CD30	302.7 (±214.5)	30.8 (13.3–47.8)	35.7 (±31.8)
CD56	17.8 (±24.6)	0.7 (0.3–2.1)	2.0 (±3.3)
CD138	12.6 (±16.8)	1.5 (0.9–3.1)	2.9 (±3.7)
Bcl-2	231.2 (±124.3)	31.2 (17.5–43.9)	51.7 (±24)
Bcl-6	30.8 (±52.3)	4.3 (0.3–7.4)	4.9 (±6.2)
Other parameters			
CD4/CD8 ratio ^b^	0.9 (0.5–1.8)		

Abbreviations: SD, standard deviation; IQR, interquartile range; NA, not applicable; CD, cluster of differentiation; CD3, pan-T-cell marker; CD4, marker of CD4 positive T-cells; CD8, marker of CD8 positive T-cells; CD20, B-cell marker; CD138, plasma cells; CD30, CD56, Bcl-2 and Bcl-6, other immunohistochemical markers. ^a^ Total number of HS patients: 49; ^b^ median (IQR); calculated ratio in skin tissue; absolute number of patients, n (%).

## Data Availability

The raw data supporting the conclusions of this article will be made available by the authors, without undue reservation.

## Appendix A

**Table A1:** Analysis of the expression of the different markers in patients with and without a positive family history of H, in patients with mild–moderate [Hurley I and II] vs. severe [Hurley III] HS disease severity and male vs. female HS patients.

*H*-Score Quantification	Positive Family History of HS	Negative Family History of HS	*p* Value	Patients with Severe Disease Severity [Hurley III]	Patients with Mild–Moderate Disease Severity [Hurley I and II]	*p* Value	Male HS Patients	Female HS Patients	*p* Value
CD3, mean (±SD) ^a,1^	64.1 (±26.5)	71.1 (±25.7)	0.2	70.6 (±25.8)	67.0 (±26.2)	0.6	69.7 (±27.5)	67.9 (±23.5)	0.8
CD4, mean (±SD) ^b,1^	31.3 (±24.6)	28.6 (±17.0)	0.7	30.3 (±21.5)	28.3 (±16.5)	0.7	31.8 (±20.9)	25.4 (±15.9)	0.3
CD8, mean (±SD) ^c,1^	30.7 (±19.2)	36.2 (±17.3)	0.3	33.0 (±18.4)	36.7 (±17.2)	0.5	35.4 (±19.9)	33.0 (±14.2)	0.7
CD20, mean (±SD) ^d,1^	52.6 (±26.6)	59.4 (±31.2)	0.5	58.2 (±29.9)	56.2 (±30.4)	0.8	64.5 (±31.5)	53.1 (±28.4)	0.2
CD30, mean (±SD) ^a,1^	31.8 (±25.7)	39.4 (±33.6)	0.5	40.9 (±38.3)	32.3 (±19.0)	0.4	36.3 (±32.4)	37.9 (±31.6)	0.9
CD56, mean (±SD) ^a,1^	1.7 (±1.6)	2.2 (±3.8)	0.6	2.4 (±3.4)	1.6 (±3.2)	0.4	2.2 (±2.9)	1.9 (±4.0)	0.8
CD138, mean (±SD) ^a,1^	1.3 (±1.6)	1.6 (±1.3)	0.5	1.4 (±1.6)	1.8 (±1.0)	0.3	1.5 (±1.4)	1.7 (±1.5)	0.7
Bcl-2, mean (±SD) ^a,1^	49.7 (±29.2)	55.6 (±20.2)	0.4	57.5 (±23.5)	49.0 (±21.5)	0.2	60.1 (±24.3)	43.8 (±17.2)	0.017 *
Bcl-6, mean (±SD) ^c,1^	4.8 (±4.9)	5.6 (±6.9)	0.7	4.1 (±4.3)	7.0 (±8.1)	0.1	3.8 (±4.1)	6.3 (±7.3)	0.2
Ki67, mean (±SD) ^b,1^	8.1 (±8.2)	11.5 (±11.5)	0.3	8.0 (±6.6)	13.7 (±13.8)	0.093	8.7 (±10.4)	11.6 (±10.8)	0.4
CD4/CD8 ratio, median (IQR) ^c,2^	1.1 (0.7–3.5)	0.9 (0.6–1.5)	0.2	0.9 (0.6–2.4)	1.1 (0.8–1.2)	0.8	1.0 (0.6–2.1)	1.1 (0.6–1.2)	0.6

Abbreviations: SD, standard deviation; d, days; IQR, interquartile range; * significant result; CD, cluster of differentiation; CD3, pan-T-cell marker; CD4, marker of CD4 positive T-cells; CD8, marker of CD8 positive T-cells; CD20, pan-B-cell marker; CD30, expression in activated T-cells and B-cells; CD56, expression of natural killer cells; Bcl-2, expression in B and T-cells and promotes anti-apoptosis; Bcl-6, expression in activated germinal centre B-cells and T follicular helper cells; Ki67, cell proliferation marker. ^1^ Statistical method: independent-samples *t* test, two-sided test (parametric). ^2^ Statistical method: Mann–Whitney U-test (non-parametric). ^a^ Total number of HS patients: 47, ^b^ Total number of HS patients: 48, ^c^ Total number of HS patients: 45, ^d^ Total number of HS patients: 46.

## Appendix B

**Table A2:** Analysis of marker expression between HS patients with vs. without obesity [BMI ≥ 30 kg/m^2^] in HS patients with vs. without hypertension and with vs. without diabetes mellitus.

*H*-Score Quantification	HS Patients with Obesity	HS Patients Without Obesity	*p* Value	HS Patients with Hypertension	HS Patients Without Hypertension	*p* Value	HS Patients with Diabetes Mellitus	HS Patients Without Diabetes Mellitus	*p* Value
CD3, mean (±SD) ^a,1^	74.5 (±31.8)	65.6 (±21.2)	0.25	63.4 (±24.9)	72.1 (±26.2)	0.3	71.8 (±25.7)	68.4 (±26.1)	0.7
CD4, mean (±SD) ^b,1^	35.1 (±21.1)	23.4 (±14.2)	0.093	25.2 (±16.8)	32.0 (±20.5)	0.2	35.8(±14.7)	28.0 (±20.0)	0.3
CD8, mean (±SD) ^c,1^	32.1 (±17.4)	38.2 (±18.4)	0.3	33.2 (±19.2)	35.2 (±17.4)	0.7	39.4 (±19.2)	33.3 (±17.6)	0.4
CD20, mean (±SD) ^d,1^	58.2 (±30.8)	56.0 (±29.0)	0.8	49.4 (±26.0)	62.0 (±31.3)	0.2	45.9 (±24.9)	60.1 (±30.5)	0.2
CD30, mean (±SD) ^a,1^	39.7 (±30.4)	33.4 (±33.8)	0.5	37.3 (±28.3)	37.2 (±27.7)	>0.9	42.6 (±48.1)	36.0 (±27.0)	0.6
CD56, mean (±SD) ^a,1^	2.6 (±4.0)	1.2 (±1.4)	0.2	1.4 (±1.3)	2.4 (±4.0)	0.3	3.1 (±4.6)	1.8 (±3.0)	0.3
CD138, mean (±SD) ^a,1^	1.5 (±1.4)	1.7 (±1.5)	0.6	1.5 (±1.5)	1.6 (±1.3)	0.8	1.6 (±1.3)	1.5 (±1.4)	0.8
Bcl-2, mean (±SD) ^a,1^	53.3 (±23.6)	54.8 (±23.0)	0.8	53.2 (±24.3)	54.3 (±22.8)	0.9	58.5 (±21.1)	52.8 (±23.5)	0.5
Bcl-6, mean (±SD) ^c,1^	5.5 (±5.6)	5.1 (±7.4)	0.9	4.9 (±4.9)	5.6 (±7.0)	0.7	8.7 (±6.8)	4.6 (±6.0)	0.1
Ki67, mean (±SD) ^b,1^	10.8 (±10.8)	10.0 (±10.7)	0.8	13.0 (±12.4)	9.0 (±9.4)	0.2	11.9 (±6.8)	10.2 (±11.4)	0.7
CD4/CD8 ratio, median (IQR) ^c,2^	1.2 (0.8–2.3)	0.7 (0.5–1.1)	0.014 *	0.9 (0.5–2.1)	1.1 (0.7–1.6)	0.6	1.1 (0.8–2.4)	1.1 (0.6–1.8)	0.5

Abbreviations: SD, standard deviation; d, days; IQR, interquartile range; *, significant result; CD, cluster of differentiation; CD3, pan-T-cell marker; CD4, marker of CD4 positive T-cells; CD8, marker of CD8 positive T-cells; CD20, pan-B-cell marker; CD30, expression in activated T-cells and B-cells; CD56, expression of natural killer cells; Bcl-2, expression in B and T-cells and promotes anti-apoptosis; Bcl-6, expression in activated germinal centre B-cells and T follicular helper cells; Ki67, cell proliferation marker. ^1^ Statistical method: independent-samples *t* test, two-sided test (parametric). ^2^ Statistical method: Mann–Whitney-U-Test (non-parametric). ^a^ Total number of HS patients: 47, ^b^ Total number of HS patients: 48, ^c^ Total number of HS patients: 45, ^d^ Total number of HS patients: 46.

## Appendix C

**Table A3:** Analysis of the CD4/CD8 ratio in lesional skin tissue of HS patients.

Parameter	CD8 Dominance (CD4/CD8 Ratio < 1)	CD4 Dominance (CD4/CD8 Ratio > 1)	*p* Value
Absolute number (%)	21 (46.7)	24 (53.3)	NA
Age ^a^	55 (39–59)	40.5 (34.8–53.8)	0.2
Age of HS onset ^a^	30 (20–38)	25.5 (19–31.3)	0.6
Duration of HS ^a^	15 (8–27)	11 (7–24.3)	0.5
Male ^b^	7 (33.3)	10 (41.7)	0.6
BMI ^a^	29.7 (26.9–32.4)	35.0 (30.2–36.8)	0.05
Height in m ^a^	1.77 (1.73–1.8)	1.72 (1.65–1.81)	0.3
Weight in kg ^a^	90 (78–100)	101.5 (90.5–118.8)	0.076
Active smoker ^b^	13 (72.2)	17 (73.9)	0.9
Tobacco pack-years ^a,e^	20 (0–31.8)	15 (0–23.5)	0.5
Number of inflammatory nodules ^a^	1 (0-3)	2 (1–4.3)	0.3
Number of abscesses ^c^	0 (0–4)	0 (0–0)	0.027 *
Number of fistulas ^a^	6 (3–11)	5.5 (2–10)	0.3
Flare-ups in the last 4 weeks ^a^	0 (0–1)	0.5 (0–3)	0.075
Current pain ^a^	3 (0–5)	2 (0–6)	0.8
mHSS ^a^	50 (28–90)	49.5 (24–76.5)	0.5
Hurley III ^b^	14 (66.7)	13 (54.2)	0.4
SAHS ^a^	8 (5–8)	8 (5.8-10)	0.5
Hypertension ^b^	9 (42.9)	7 (29.2)	0.3
Diabetes mellitus ^b^	4 (19.0)	5 (20.8)	0.9
Hypothyroidism ^b^	6 (24)	1 (4.8)	0.062
Dyslipidaemia ^b^	2 (9.5)	4 (16.7)	0.5
Axillary involvement ^b,d^	16 (76.2)	16 (66.7)	0.5
Mammary involvement ^b,d^	0 (0)	2 (8.3)	0.2
Abdominal involvement ^b,d^	2 (9.5)	2 (8.3)	0.9
Mons pubis involvement ^b,d^	6 (28.6)	15 (62.5)	0.023 *
Genital involvement ^b,d^	7 (33.3)	8 (33.3)	1
Inguinal involvement ^b,d^	11 (52.4)	15 (62.5)	0.5
Thigh involvement ^b,d^	3 (14.3)	4 (16.7)	0.8
Perianal involvement ^b,d^	6 (28.6)	5 (20.8)	0.5
Gluteal involvement ^b,d^	8 (38.1)	5 (20.8)	0.2

Abbreviations: n, absolute number of patients; y, years; BMI, body mass index; IQR, interquartile range; mHSS, modified Hidradenitis Suppurativa Score; HS, Hidradenitis suppurativa; SAHS, Severity Assessment of Hidradenitis Suppurativa; mHSS, modified Hidradenitis Suppurativa Score; NA, not applicable; CD, cluster of differentiation; CD4, marker of CD4 positive T-cells; CD8, marker of CD8 positive T-cells. *p* value: * significant result (*p* < 0.05). ^a^ Calculated with Mann–Whitney-U test. Data given as median (IQR), ^b^ Calculated with Chi-square test. Data given as absolute number of patients (n) in %, ^c^ Calculated with Mann–Whitney-U test. Data given as median (range), ^d^ Location refers to the skin area of the anatomical region; ^e^ The number of pack-years is calculated by multiplying the number of cigarettes smoked per day by the number of years smoked. A pack is defined as 20 cigarettes per pack.

## Appendix D

**Table A4:** Analysis of B-cell-dominated HS in contrast to T-cell-dominated HS.

Parameter	B-Cell-Dominated HS (Absolute Number of Sum of CD20+ and CD138+ Cells > CD3+ Cells)	T-cell-Dominated HS (Absolute Number of CD3+ Cells > Sum of CD20+ and CD138+ Cells)	*p* Value
Absolute number (%)	15 (33.3)	30 (66.7)	NA
Age ^a^	43 (32.5–49.5)	54 (36–57)	0.3
Age of HS onset ^a^	23 (18–30)	27.5 (20–37.8)	0.2
Duration of HS ^a^	11 (6–24.5)	12 (8–25.8)	0.5
Male ^b^	8 (53.3)	9 (30.0)	0.1
BMI ^a^	36.1 (29.7–37.0)	31.1 (27.8–36.3)	0.5
Height in m ^a^	1.75 (1.65–1.78)	1.75 (1.68–1.82)	0.4
Weight in kg ^a^	100 (85–111.5)	99 (85.3–117)	0.9
Active smoker ^b^	7 (58.3)	23 (79.3)	0.2
Tobacco pack-years ^a,e^	5 (0–18.8)	20 (2.8–30)	0.06
Number of inflammatory nodules ^a^	3 (1–5)	1 (0–3)	0.071
Number of abscesses ^c^	0 (0-4)	0 (0–1)	0.2
Number of fistulas ^a^	4 (3–8.5)	6 (2.3–11)	0.7
Flare-ups in the last 4 weeks ^a^	0 (0–1)	0 (0–2)	0.4
Current pain ^a^	3 (0.5–5.5)	2 (0–5.8)	0.8
mHSS ^a^	39 (30–65.5)	56.5 (21–89.8)	0.8
Hurley III ^b^	7 (46.7)	19 (63.3)	0.3
SAHS ^a^	8 (6.5–8)	8 (5–9)	0.6
Hypertension ^b^	4 (26.7)	12 (40.0)	0.4
Diabetes mellitus ^b^	1 (6.7)	7 (23.3)	0.2
Hypothyroidism ^b^	2 (13.3)	5 (16.7)	0.8
Dyslipidaemia ^b^	2 (13.3)	3 (10.0)	0.7
Axillary involvement ^b,d^	10 (66.7)	22 (73.3)	0.6
Mammary involvement ^b,d^	2 (13.3)	0 (0)	0.041 *
Abdominal involvement ^b,d^	2 (13.3)	2 (6.7)	0.5
Mons pubis involvement ^b,d^	7 (46.7)	13 (43.3)	0.8
Genital involvement ^b,d^	3 (20.0)	11 (36.7)	0.3
Inguinal involvement ^b,d^	8 (53.3)	17 (56.7)	0.8
Thigh involvement ^b,d^	4 (26.7)	3 (10.0)	0.1
Perianal involvement ^b,d^	3 (20.0)	8 (26.7)	0.6
Gluteal involvement ^b,d^	7 (46.7)	6 (20.0)	0.063

Abbreviations: n, absolute number of patients; y, years; BMI, body mass index; IQR, interquartile range; mHSS, modified Hidradenitis Suppurativa Score; HS, Hidradenitis suppurativa; SAHS, Severity Assessment of Hidradenitis Suppurativa; mHSS, modified Hidradenitis Suppurativa Score; NA, not applicable; CD, cluster of differentiation; CD3, pan-T-cell marker; CD20, B-cell marker; CD138; marker for plasma cells. *p* value: * significant result (*p* < 0.05). ^a^ Calculated with Mann–Whitney-U test. Data given as median (IQR), ^b^ Calculated with Chi-square test. Data given as absolute number of patients (n) in %, ^c^ Calculated with Mann–Whitney-U test. Data given as median (range), ^d^ Location refers to the skin area of the anatomical region; ^e^ The number of pack-years is calculated by multiplying the number of cigarettes smoked per day by the number of years smoked. A pack is defined as 20 cigarettes per pack.
